# Effect of the short-segment internal fixation with intermediate inclined-angle polyaxial screw at the fractured vertebra on the treatment of Denis type B thoracolumbar fracture

**DOI:** 10.1186/s13018-020-01686-7

**Published:** 2020-05-24

**Authors:** Chengjie Xiong, Biwang Huang, Tanjun Wei, Hui Kang, Feng Xu

**Affiliations:** 1 Orthopaedic Department, General Hospital of Central Theater Command of PLA, #627 Wuluo Road, Wuchang District, Wuhan, Hubei China; 2 Wuhan University of Science and Technology, Wuhan, China

**Keywords:** Thoracolumbar fracture, Short-segment internal fixation, Long-segment internal fixation, Inclined-angle screw placement, Intermediate screw

## Abstract

**Background:**

Short-segment internal fixation with intermediate straight-forward monoaxial screws (SSIF-SFM) and long-segment internal fixation (LSIF) are the two major surgical options for thoracolumbar (TL) fracture. However, SSIF-SFM might not provide adequate support to the spine, and LSIF is unnecessarily extensive. SSIF with intermediate inclined-angle polyxial screw (SSIF-IAP) might offer an alternative solution for the treatment of TL fracture.

**Methods:**

A retrospective study was conducted. Sixty-nine patients (47 males and 22 females; average 34.5 years) with Denis type B TL fracture who met the criteria for inclusion were enrolled. Sagittal Cobb’s angle (SCA), anterior vertebral body height (AVBH), vertebral body index (VBI), and spinal canal encroachment (SCE) were measured and assessed. Visual analogue scale (VAS) and Oswestry disability index (ODI) were also evaluated.

**Results:**

The average values of incision length, blood loss, duration of operation, and hospital stay in the SSIF-IAP group and SSIF-SFM group were significantly decreased compared with those in the LSIF group. The AVBH and VBI in the SSIF-IAP group and LSIF group were significantly improved than those in the SSIF-SFM group at 6-month and the latest follow-ups (*P* < 0.05). The correction losses of AVBH and VBI (calculated by the reduction of AVBH and VBI) in the SSIF-IAP group and LSIF group were also significantly decreased compared with those in the SSIF-SFM group at 6-month and the latest follow-ups (*P* < 0.05). There was no significant difference of SCE among the three groups postoperatively. The VAS and ODI in the SSIF-IAP group and SSIF-SFM group were significantly decreased compared with those in the LSIF group at 6-month and the latest follow-ups (*P* < 0.05).

**Conclusion:**

Both SSIF-IAP and LSIF can improve the biomechanical stability as compared with SSIF-SFM. Moreover, SSIF-IAP was less extensive compared to LSIF. SSIF-IAP was an effective and reliable operative technique for patients with Denis type B TL fracture.

## Introduction

The thoracolumbar (TL) junction is a transition zone between the rigid thoracic spine and the more mobile lumbar spine (from T11 to L2), and nearly 70% of all traumatic spinal injuries occur within this region [1, 2]. Treatment of TL fracture remains controversial, especially in patients without severe neurological symptoms. Although conservative treatment is often recommended in the majority of patients, clinical studies have demonstrated that surgical treatment can lead to better fracture reduction, stronger internal fixation, and more favorable long-term clinical outcomes [3, 4]. An isolated posterior approach for surgical treatment of TL fracture is often preferred [5, 6]. There are two main reasons for this choice. Firstly, more postoperative complications are associated with an anterior approach as compared with the posterior approach [7–9]. In addition, spine surgeons are more familiar with the posterior approach due to its easier application [10].

Short-segment internal fixation (SSIF) via the posterior approach is the most common treatment for TL fracture [11]. Although SSIF can obtain satisfactory reduction, it often leads to instrumentation failure due to osteoporosis and correction loss [12]. Long-segment internal fixation (LSIF) is an alternative solution, which can increase construct stiffness and reduce the load on each screw by application of long segmental instrumentation; however, LSIF is unnecessarily extensive and decreases the number of motion segments. In addition, LSIF is often associated with the development of adjacent-segment degeneration (ASD) disease [13–15]. Saving the motion segments is an important principle of spine surgery. Therefore, in order to restrict the number of fusion segments and improve instrumentation efficiency, additional pedicle screws at the fracture level are applied along with SSIF to treat TL fracture [16, 17]. Intermediate screws in the SSIF system are usually paralleled with the superior endplate with relatively short length [18]. However, as a result of collapse of anterior and middle columns in TL fracture, SSIF with straight-forward monoaxial screws cannot always provide sufficient biomechanical support for the anterior column [19].

Recently, biomechanical studies [20, 21] have shown that relatively long length pedicle screws could significantly increase the pullout force of the screws and the stiffness of the internal fixation system. We subsequently modified the traditional SSIF with straight-forward monoaxial screws and developed a new technique for TL fracture. We changed the direction of implementation of pedicle trajectory as compared with the standard implementation, so longer polyaxial pedicle screws can be implanted with an inclined angle at the fracture level. These intermediate inclined-angle polyaxial screws can help stabilize the anterior and middle columns of the fractured vertebra, which would finally improve the stability of SSIF system [22, 23]. Therefore, a retrospective study was conducted to compare the feasibility, safety, and efficacy of SSIF with inclined-angle polyaxial screws (SSIF-IAP) with SSIF with straight-forward monoaxial screws (SSIF-SFM) and LSIF with straight-forward monoaxial screws for the treatment of Denis type B TL fracture.

## Material and methods

### Patient population

**Fig. 1:**
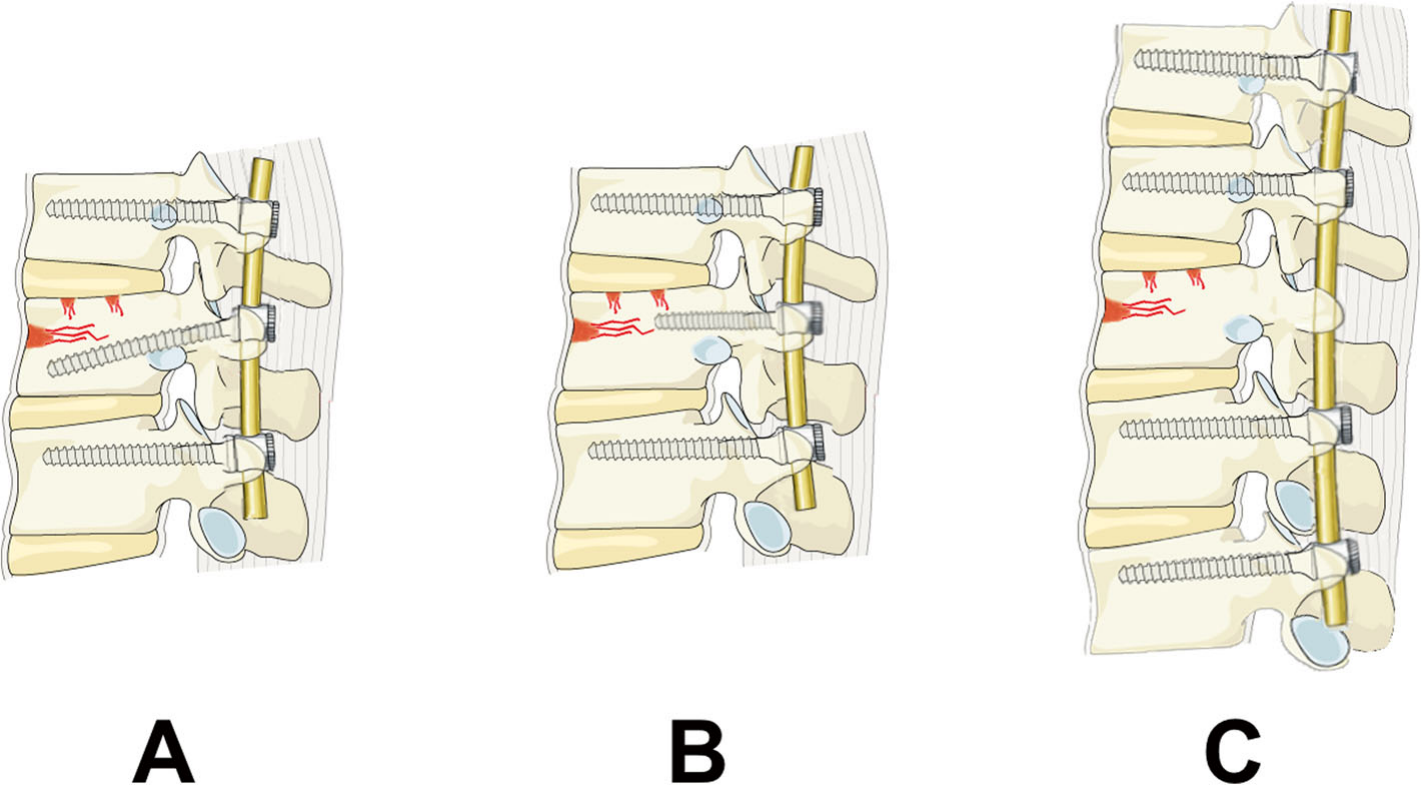
The illustration of three different fixations for the treatment of Denis type B TL fracture. **a** Inclined-angle screws were inserted into the fracture vertebra via the posterior approach along with SSIF. **b** Straight-forward screws were inserted into the fracture vertebra via the posterior approach along with SSIF. **c** Two pedicle screws above and below the fracture vertebra were inserted via the posterior approach by application of LSIF. TL, thoracolumbar; SSIF, short-segment internal fixation; LSIF, long-segment internal fixation

Seventy-eight patients met the criteria for inclusion. Five patients refused the surgical treatment, and were lost to follow-up. Sixty-nine patients (47 males and 22 females), ranging from 22 to 54 years old (average 34.5 years), were enrolled in this study. Only patients with a single-level TL junction (T11–L2) fracture were included and received surgical management in our department between May 2011 and May 2015. Patients were divided into three groups: (1) SSIF-IAP: SSIF with intermediate inclined-angle polyaxial screws at the fracture level; (2) SSIF-SFM: SSIF with intermediate straight-forward monoaxial screws at the fracture level; and (3) LSIF: long-segment internal fixation using two monoaxial pedicle screws above and below the fracture level (Fig. 1).

The study was approved by the Ethics Committee of General Hospital of Central Theater Command and was in accordance with the Helsinki Declaration.

### Inclusion and exclusion criteria

The inclusion criteria were (i) Denis type B TL fracture, (ii) no dislocation fracture, (iii) absence of obvious neurological impairment (Frankel grades A and B), and (iv) from trauma to operation being less than 1 week. The exclusion criteria were (i) the fractured inferior endplate that was confirmed by computed tomography (CT) scans, (ii) pathologic fracture, (iii) osteoporotic fracture, (iv) bilateral pedicle fracture, (v) previous spinal surgery history, (vi) other major organ system injuries, and (vii) pregnancy.

### Surgical procedures

After induction of general anesthesia with endotracheal intubation, each patient was placed in the prone position on a specialized operating frame that both shoulders and superior iliac spines were supported by gel pads to create hyperextension position of the spine and achieve postural reduction. In the SSIF-IAP group, after determination of the fracture level using fluoroscopy, a midline vertical skin incision was made to strip the erector spinae muscles bilaterally, and spinous processes and laminae were then exposed. Four pedicle screws were bilaterally implanted into adjacent vertebrae above and below the fractured vertebra. For the fractured vertebra, hemi-laminectomy or laminectomy was performed, and then an “L”-shaped chisel was inserted into the fractured vertebra to reduce the compressed and fractured vertebra in the spinal canal. The superior endplate was injured, and the inferior endplate was intact in the Denis type B TL fracture. The starting point was 2 mm superior to the standard landmark, and the insertion of pedicle screws was approximately 10° to 20° inclined to the inferior endplate. Unilateral or bilateral pedicle screws were implanted according to the integrity of the pedicle of the fractured vertebra. The inclined-angle pedicle screws were purchased in the residual lower portion of the injured vertebral body. After all pedicle screws were attached, two rods were applied to connect pedicle screws on both sides using the rod placement system. The reduction and fixation were confirmed by fluoroscopy, and the incision was then irrigated and sutured. All operations were performed by the same surgery group. The procedure for LSIF and SSIF-SFM was described as previously [16]. Only instrumentation without bone graft was utilized. If no spinal canal compression was observed before operation, pedicle screws can also be implanted percutaneously under fluoroscopic guidance. The internal fixation stabilization system was supplied by Shandong Weigao Company of China.

All patients were routinely administered prophylactic antibiotics postoperatively for 48 h, and sterile dressing of incision was replaced every 2 days until the suture was removed. Patients were encouraged to start physical activities under the protection of brace; however, excessive and heavy activities were restricted up to 12 weeks after the operation. Following discharge from the hospital, patients were clinically and radiologically assessed at monthly intervals in the orthopedic outpatient clinic, with a mean follow-up of 24.01 months (range, 18–36 months).

### Clinical assessment

Radiographic evaluation consisted of sagittal Cobb’s angle (SCA), anterior vertebral body height (AVBH), vertebral body index (VBI), and spinal canal encroachment (SCE). The SCA, AVBH, and VBI were measured as previously described [17]. SCE was obtained from serial transverse CT scans by using ImageJ (NIH, Bethesda, MD) on admission and immediately after surgery, and it was used to evaluate the extent of the spinal canal decompression [24]. The correction losses of AVBH were calculated from the equation: postoperative AVBH (1 week) − postoperative AVBH (6 months or the latest)/postoperative AVBH (1 week). The correction losses of VBI were calculated from the equation: postoperative VBI (1 week) − postoperative VBI (6 months or the latest)/postoperative VBI (1 week). All data were analyzed by an independent observer who was not involved in the treatment of patients.

The clinical data from all included patients were obtained and assessed. Visual analogue scale (VAS) and Oswestry disability index (ODI) questionnaires were used to evaluate functional status preoperatively and at each follow-up time point (1-week, 6-month, and the latest follow-up). The VAS and ODI scores were recorded in the questionnaires at each follow-up in the orthopedic outpatient clinic.

### Statistical analyses

The Kruskal-Wallis test, chi-square test, Wilcoxon test (dependent data), and Mann-Whitney *U* test (independent data) were performed to analyze the variables using the SPSS 17.0 software (SPSS Inc, Chicago, USA). Quantitative data were represented as the median (range) and a statistically significant difference with *P* value < 0.05. No alpha adjustment for multiple testing was performed.

## Results

### Demographic characteristics

Baseline demographic data including age, sex, body mass index (BMI), cause of injury, fracture site, and neurological status in the three groups were collected and compared. No significant differences were observed among the three groups regarding the demographic data. The injured vertebral segments were T11 in 7 cases, T12 in 14 cases, L1 in 31 cases, and L2 in 17 cases. The fractures were caused by traffic accidents in 25 cases, falling from height in 30 cases, and other accidents in 14 cases. There were 7 cases of Frankel grade C, 22 cases of Frankel grade D, and 40 cases of Frankel grade E (Table 1).

**Table 1:**
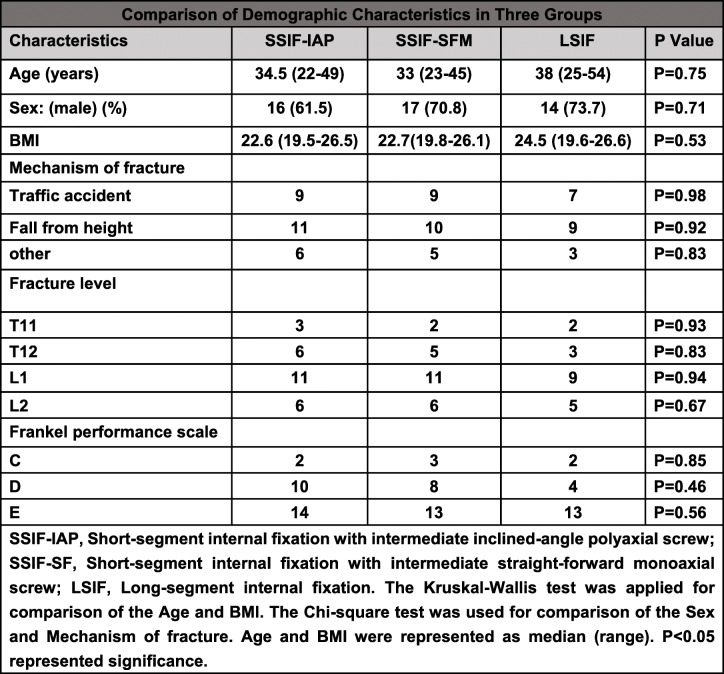
Comparison of demographic characteristic in three groups

The average incision length was 10.4 cm (7.2–16.3 cm). The mean blood loss was 110 ml (30–450 ml) during operation. The average duration of operation was 122.4 min (98–155 min). The average hospital stay was 14.8 days (10–22 days). There were no significant differences between the SSIF-IAP group and the SSIF-SFM group with regard to incision length, mean blood loss, duration of operation, and hospital stay. The average values of these parameters in the SSIF-IAP group and SSIF-SFM group were significantly decreased compared with those in the LSIF group (Table 2).

**Table 2:**
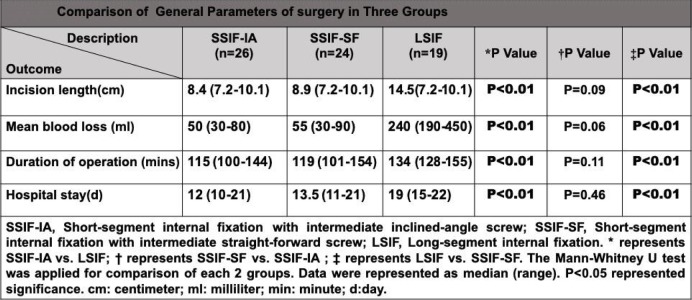
Comparison of general parameters of surgery in three groups

### Radiologic outcomes

Significant improvements in SCA, AVBH, VBI, and SCE of the fractured vertebra following the operation were observed among the three groups (*P* < 0.01) (Supplementary Table 1). There were no significant differences between the SSIF-IAP group and SSIF-SFM group with regard to SCA postoperatively (*P* > 0.05); however, SCA in the LSIF group was significantly improved than that in the SSIF-SF group at 6-month follow-up (*P* < 0.05). Improvements in AVBH and VBI were not in agreement with SCA postoperatively. AVBH in the SSIF-IAP group and LSIF group were significantly improved than those in the SSIF-SFM group at 6-month and the latest follow-ups (*P* < 0.05). VBI in the SSIF-IAP group and LSIF group were also significantly improved than those in the SSIF-SFM group at 6-month and the latest follow-ups (*P* < 0.05). There were no significant differences among the three groups with regard to SCE postoperatively (*P* > 0.05) (Table 3).

**Table 3:**
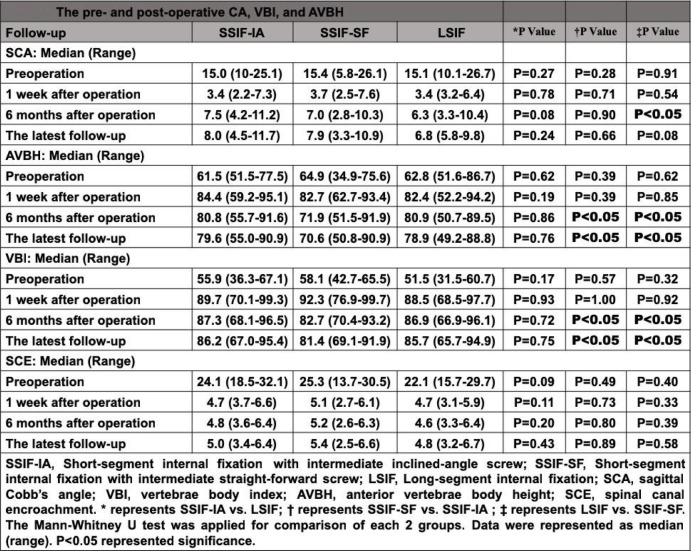
The pre-and post-oprative CA, VBI and AVBH

Similar to the results mentioned above, there were no significant differences among the three groups with regard to the correction losses of SCA (*P* > 0.05). However, the correction losses of AVBH in the SSIF-IAP group and LSIF group were significantly decreased compared with those in the SSIF-SFM group (*P* < 0.05); the correction losses of VBI in the SSIF-IAP group and LSIF group were also significantly decreased compared with those in the SSIF-SFM group (*P* < 0.05) (Table 4).

**Table 4:**
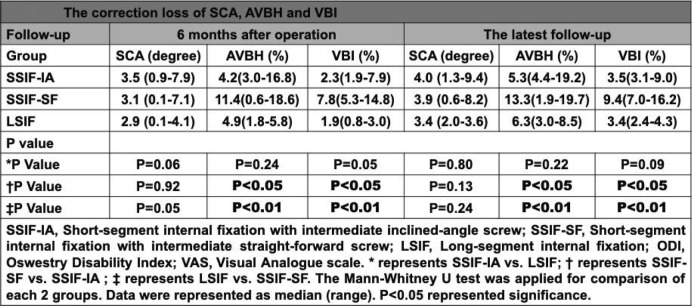
The correction loss of SCA, AVBH and VBI

### Clinical outcomes

The VAS score following operation was significantly improved in the three groups (*P* < 0.01) (Supplementary Table 2). There were no significant differences among the three groups with regard to the VAS score at the pre-operation and 1-week follow-ups (*P* > 0.05); however, the VAS scores in the SSIF-IAP group and SSIF-SFM group were significantly decreased compared with those in the LSIF group at the 6-month and the latest follow-ups (*P* < 0.05). The ODI score following the operation was also significantly improved in the three groups (*P* < 0.01) (Supplementary Table 2). Similarly, there were no significant differences among the three groups with regard to the ODI score at the pre-operation and 1-week follow-ups (*P* > 0.05); however, the ODI scores in the SSIF-IAP group and SSIF-SFM group were significantly decreased compared with those in the LSIF group at the 6-month and the latest follow-ups (*P* < 0.05) (Table 5).

**Table 5:**
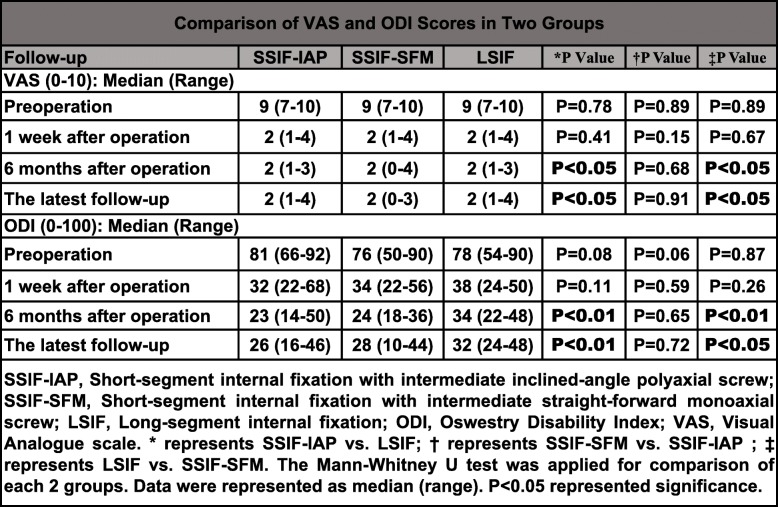
Comparison of VAS and ODI scores in two groups

### Complications

No major complications, such as nerve injury, wound infections, and non-fusion, occurred among the three groups postoperatively. There was one case of screw breakage at the 6-month follow-up, with a 4.16% failure rate in the SSIF-SFM group; there was one case of screw loosing at the 1-year follow-up, with a 5.26% failure rate in the LSIF group. However, there was no significant difference between the two groups regarding the failure rate (*P* = 0.87). Both patients underwent conservative treatment until solid bony fusion of the superior vertebral body was observed, and then the implant was removed. All patients with incomplete neurological impairment in the three groups improved at least one grade according to the Frankel performance scale. Only minor neurological impairment (Frankel grades D or E) was found at the latest follow-up in all patients.

### Representative cases

**Fig. 2:**
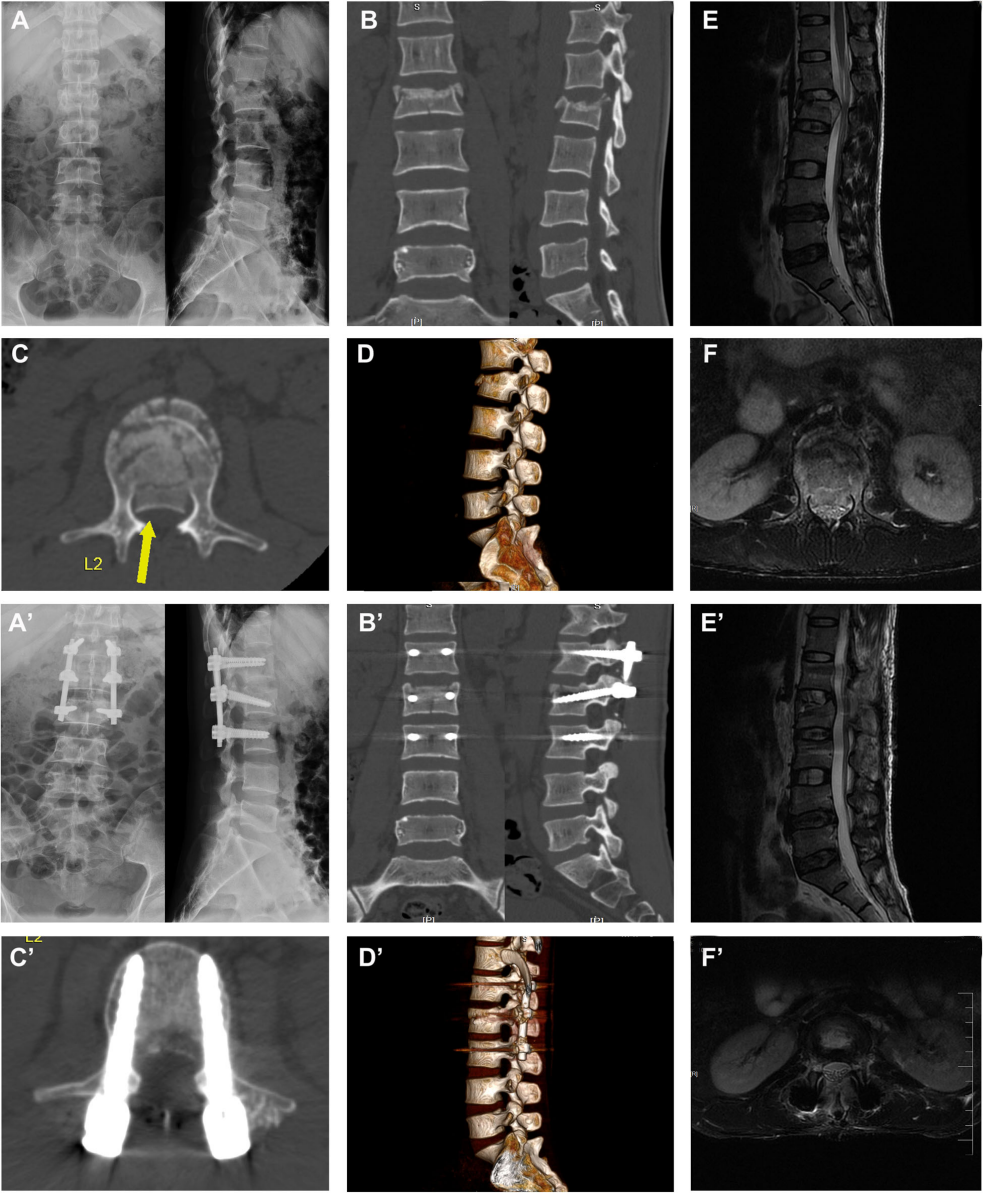
X-ray, CT, and MRI of a case with Denis type B TL fracture treated by SSIF-IA system. **A**, **A**’ Anteroposterior and lateral X-ray demonstrating the L2 compression fracture before and after operation. **B**, **B**’ Lateral spiral CT showing the L2 compression fracture with injured superior endplate before and after operation. **C**, **C**’ Axial spiral CT showing the spinal canal encroachment by fragments of the fractured vertebra before and after operation. **D**, **D**’ The 3D reconstruction of lumbar spine with the L2 compression fracture before and after operation. **E**, **E**’ Sagittal MRI confirming the L2 compression fracture with vertebra edema before and after operation. **F**, **F**’ Axial MRI confirming the spinal canal encroachment by fragments of the fracture vertebra along with posterior elements of the vertebra before and after operation. CT, computed tomography; MRI, magnetic resonance imaging; 3D, 3-dimensional

Representative case who underwent an operation via inclined-angle screw placement is presented in Fig. 2.

## Discussion

Although conservative management is thought to be the optimal treatment for TL junction fracture without severe neurological impairment, it is often accompanied by discomfort and limited mobility. Surgical intervention is therefore preferred in patients with TL junction fracture, because it can maintain reduction, prevent further deformity and neurologic deterioration, and improve mobilization. Especially for young patients, surgical intervention may have advantageous effects for the recovery of spine sagittal alignment in the long run. The selection of the surgical approach in the management of TL junction fracture is dependent on many variables, such as bone intensity, kyphotic deformity, and spinal canal encroachment. Either the isolated anterior/posterior approach or the combined approach can be applied for the stabilization of unstable spine. Studies have shown that the anterior instrumentation with bone graft can provide reliable internal fixation, but it is a more invasive approach that is associated with complications and prolonged postoperative recovery [8, 25]. Alternative intervention is considered prior to the anterior approach. LSIF via the posterior approach can also improve and maintain optimal stability of the spinal column; however, it might decrease spinal range of motion and increase the incidence of ASD. Therefore, other improved alternatives have been lately developed to minimize its adverse effects.

Superior biomechanical stability is found in SSIF with addition of pedicle screws at the fracture level without sacrificing benefits of SSIF. Studies have shown that SSIF with intermediate screws could significantly improve the biomechanical stability and construct stiffness as compared with SSIF [11, 26]. Moreover, clinical research has found that the restoration of the fractured vertebral height obtained in SSIF with intermediate screws was equivalent to that in LSIF [18]. Secondly, intermediate screws at the fracture level can optimize load on the instrumentation system and reduce the risk of broken screws or rods. Post-buckling of the rod is more evident within the four-screw fixation construct than within the six-screw fixation construct [27]. This is due to that the rod of the four-screw fixation construct spans a longer distance between two screws as compared with the six-screw fixation construct, and tension strain at each level of the four-screw fixation construct is significantly increased compared with that at each level of the six-screw fixation construct [28, 29]. Although traditional SSIF with intermediate screws theoretically corrects kyphotic deformity, however, this instrumentation system is not able to provide adequate support to the anterior column of the fractured vertebra for unstable TL fracture in practice.

We then developed a modified SSIF with inclined-angle intermediate polyaxial screws. There are following advantages. Firstly, this inclined-angle insertion can increase the length of pedicle screws, so it can increase the pullout force and provide greater construct stiffness. Denis type B fracture is a special categorized fracture, in which the superior endplate is mainly involved, while the inferior endplate and the lower portion of the injured vertebral body usually escapes from the injury site [30]. Therefore, the residual vertebral body and the caudal disc are preserved, and they are able to tolerate anterior column reconstruction. The pedicle screws in the SSIF-IAP group were inserted into the lower residual portion of the injured vertebral body, which would contribute to the pullout strength. In addition, the “eggshell” deformity often occurred postoperatively, and the fractured vertebra cannot provide enough construct stiffness during the healing process of fracture [31]. The potential reason for the “eggshell” effect is that the vertebral height is fully restored by the internal fixation device, but the compressed bone trabeculae are not restorable, which results in a defect in the injured vertebral body [32]. To prevent this, several techniques have been developed to augment the anterior column in the unstable fractures, such as polymethylmethacrylate (PMMA) injection; however, injection of PMMA into the injured vertebral body might lead to cement extrusion, particularly when the posterior longitudinal ligament is torn [33]. Intermediate screws in the SSIF-SFM group are paralleled with the superior endplate, and the end portion of screws in the eggshell-like cavity cannot provide additional interface strength. Nevertheless, intermediate screws in the SSIF-IAP group can escape from this cavity and contribute to the interface strength. It might minimize negative effects caused by the “eggshell” deformity and promote fracture healing by increasing structural stability. However, no data are available to support this assumption that needed to be verified by further biomechanical study. During the follow-up period of over 2 years, none of the patients in the SSIF-IAP group exhibited loosening or shifting of the intermediate screws at the fracture level. The main reason for this difference might be due to that screw-to-bone interface strength was improved by the increased angulation of screws, and the anterior and middle spinal columns were immediately strengthened by these inclined-angle polyaxial screws. It suggests that inclined-angle polyaxial screws at the fracture level can protect the fractured vertebra from anterior loads and improve construct stiffness.

This retrospective study evaluated radiological outcomes of 69 patients with TL fracture who were treated with three different internal fixations. SSIF with intermediate inclined-angle screws provided better postoperative correction and maintenance compared with using SSIF with intermediate straight-forward screws. Although there was no significant difference among the three groups with regard to SCA, however, significant changes of AVBH and VBI were observed postoperatively. The initial correction of AVBH and VBI in the SSIF-IAP group was better than that in the SSIF-SFM group. Moreover, the correction losses of AVBH and VBI in the SSIF-IAP group were also significantly decreased compared with those in the SSIF-SFM group at the 6-month and the latest follow-ups. Although AVBH, VBI, and SCA are important radiological parameters for the evaluation of the fractured vertebra, however, they do not go hand in hand sometimes [34]. We attributed minor changes of SCA to the fact that intermediate inclined-angle screws might restore the height of fractured vertebra more effectively as compared with the correction of kyphotic angle, which was similar to the previous study [34]. Although the correction and maintenance of the fractured vertebral body was the best in the LSIF group, however, from a statistical point of view, the statistical difference for the correction losses between the SSIF-IAP and LSIF was not significant. In addition, we have also found that there is not a close relationship between SCE and neurological function recovery. It is due to that the postoperative neurological status is dependent on the severity of injury to the spinal cord at the moment of trauma [35]. We speculate that only patients with minor neurological impairment (Frankel grades C, D, and E) were included in our study so that all of them gradually recovered thereafter. Our data supported that SSIF-IAP was comparable to LSIF, and it also can provide improved fixation and better correction than SSIF-SFM for the treatment of TL junction fracture.

Values of all considered parameters (incision length, blood loss, surgical duration, and hospital stay) in the LSIF group were the highest among the three groups; however, no significant differences were observed between the SSIF-IAP group and SSIF-SFM group regarding these parameters. Moreover, significant improvements of functional outcomes (VAS back pain and ODI) were obtained in the SSIF-IAP group and SSIF-SFM group as compared with those in the LSIF group at the 6-month and the last follow-ups. Favorable surgical outcomes can be defined by 15% improvement in ODI score [36], and our data were consistent with this criteria. In addition, ODI score is associated with VAS and SF-36 [37]. The VAS changes might be explained by the corresponding ODI changes in our study. These results suggested that intermediate inclined-angle screw insertion at the fracture level did not significantly increase the surgical duration and the blood loss as compared with the traditional straight-forward screw insertion.

Alvine et al. [38] reported that 39% screw breakage was found and 23% reoperation was performed. McLain et al. [39] have shown that instrumentation failure incidence was more than 50%. In our series, instrumentation failure occurrence was decreased compared with that reported in these studies, one case of screw breakage in the SSIP-SFM group and one case of screw loosing in the LSIF group (instrumentation failure rate = 2.90%). One screw breakage above the fracture level was observed at the 6-month follow-up in a 28-year-old man (instrumentation failure rate = 4.16%). We attributed the reason for this instrumentation failure to the increased stress on the pedicle screw. This man had a history of heavy work without brace protection postoperatively. One screw loosing occurred at the 1-year follow-up in a 54-year-old woman (instrumentation failure rate = 5.26%). This patient was diagnosed as having osteoporosis preoperatively; however, she did not follow the doctor’s advice and take medicine against osteoporosis regularly during the follow-up.

There are still several limitations to this study. First, underlying factors such as the bone density, degree of disc degeneration, and vertebral size are variable. These confounding factors were offset by investigating three internal fixation strategies in the same specimen. Again, this clinical observation was based on data from relatively healthy strong bones (average 34.5 years), and a different picture might emerge in osteoporotic bones. In addition, this study evaluated short-term and small-population clinical outcomes, and findings may be biased. A long-term and large-scale prospective study should be performed to accurately evaluate the feasibility of this technique. Lastly, the speculation of this study was based on clinical observation, and future biomechanical research needed to be conducted to support this application.

In conclusion, SSIF-IAP can exert greater interface strength on the fractured vertebra and effectively maintain the height of the fractured vertebra compared with using SSIF-SF; SSIF-IAP can minimize the number of fused levels and promote rapid relief of lumbar back pain and early rehabilitation compared with using LSIF. Taken together, SSIF-IAP is an effective and reliable operative technique for patients with Denis type B TL fracture.

## Supplementary information

**Additional file 1: Supplementary Table 1.** The pre- and post-operative CA, VBI, and AVBH.

**Additional file 2: Supplementary Table 2.** Comparison of VAS and ODI scores in three groups.

## Data Availability

The datasets for this study are available from the corresponding author on reasonable request.

## Abbreviations

SSIF: Short-segment internal fixation
SSIF-IAP: Short-segment internal fixation with inclined-angle polyaxial screw
SSIF-SFM: Short-segment internal fixation with straight-forward monoaxial screw
LSIF: Long-segment internal fixation
TL: Thoracolumbar
SCA: Sagittal Cobb’s angle
AVBH: Anterior vertebral body height
VBI: Vertebral body index
SCE: Spinal canal encroachment
VAS: Visual analogue scale
ODI: Oswestry disability index
