# Supplementary figures and images for: Effect of the short-segment internal fixation with intermediate inclined-angle polyaxial screw at the fractured vertebra on the treatment of Denis type B thoracolumbar fracture
Source: J Orthop Surg Res. 2020 May 24;15:182. doi: 10.1186/s13018-020-01686-7 (PMC7245877; doi:10.1186/s13018-020-01686-7)

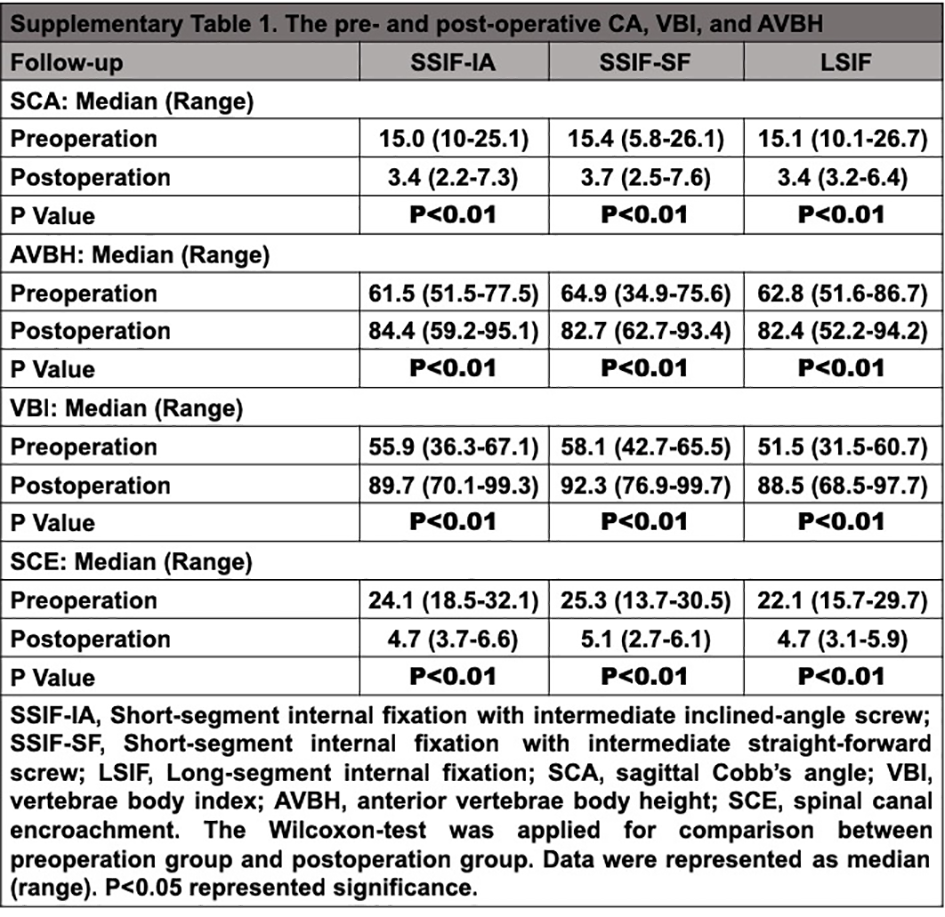

Supplement: Supplementary file 1 — Additional file 1: Supplementary Table 1. The pre- and post-operative CA, VBI, and AVBH. [file 13018_2020_1686_MOESM1_ESM.tif]

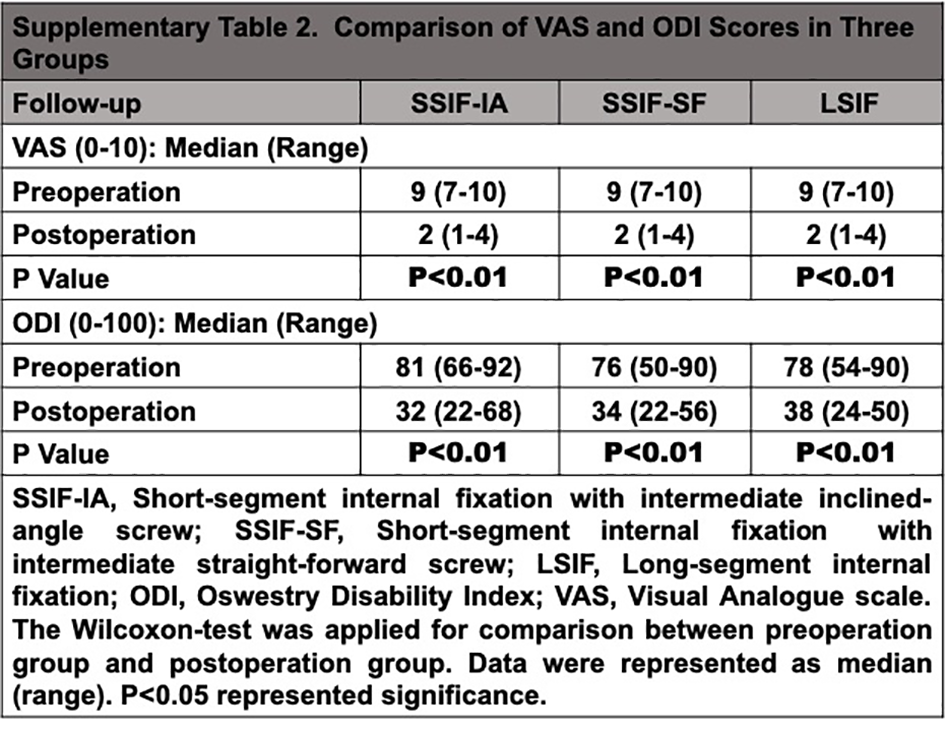

Supplement: Supplementary file 2 — Additional file 2: Supplementary Table 2. Comparison of VAS and ODI scores in three groups. [file 13018_2020_1686_MOESM2_ESM.tif]
